# An Integrated Analysis of C5AR2 Related to Malignant Properties and Immune Infiltration of Breast Cancer

**DOI:** 10.3389/fonc.2021.736725

**Published:** 2021-09-14

**Authors:** Yumeng Zhu, Xiaochao Wang, Yanqing Xu, Lu Chen, Peipei Ding, Jianfeng Chen, Weiguo Hu

**Affiliations:** ^1^Fudan University Shanghai Cancer Center and Institutes of Biomedical Sciences, Shanghai Medical College, Fudan University, Shanghai, China; ^2^State Key Laboratory of Oncology in South China, Collaborative Innovation Center of Cancer Medicine, Sun Yat-sen University Cancer Center, Guangzhou, China; ^3^Guangdong Key Laboratory of Nasopharyngeal Carcinoma Diagnosis and Therapy, Sun Yat-sen University Cancer Center, Guangzhou, China; ^4^Key Laboratory of Breast Cancer in Shanghai, Fudan University Shanghai Cancer Center, Fudan University, Shanghai, China

**Keywords:** C5AR2, breast cancer, malignant, immune infiltration, prognosis

## Abstract

**Background:**

C5AR2 (GPR77, C5L2) is the second receptor for C5a that is a potent protein generated by complement activation. C5AR2 can mediate its own signaling events and exert significant immunomodulatory effects through those events. However, research of C5AR2 in cancer is limited, and its function remains unclear in breast cancer.

**Methods:**

The expression of C5AR2 and its correlations with prognosis, immune infiltration, tumor mutation burden (TMB), and microsatellite instability (MSI) in more than thirty types of cancers were described through GTEx, TCGA, PrognoScan, TIMER2.0, CCLE, HPA, and TISIDB database. C5AR2 showed strong relationships to those immune marker sets in breast cancer. Otherwise, CCK8 assay and Transwell assay were conducted to illustrate the role of C5AR2 in migration, invasion, and proliferation of breast cancer cells.

**Results:**

Generally, C5AR2 expression differed across most cancerous and noncancerous tissues, and high C5AR2 expression significantly related to poor prognosis in BRCA, GBM, KICH, LAML, LGG, LIHC, PAAD, and STAD. Moreover, C5AR2 expression levels were dramatically correlated with recognized immune infiltration, especially the polarization of macrophages in breast cancer. Gene set enrichment analysis confirmed that C5AR2 participates in regulating multiple signaling pathways involved in tumorigenesis as well as tumor immunity. C5AR2 overexpression facilitated the functions such as migration, invasion, and proliferation in breast cancer cells, which is consistent with bioinformatics analysis.

**Conclusions:**

C5AR2 is involved in immune infiltration and malignant characteristics of breast cancer, which may be a prospective biomarker for breast cancer.

## Introduction

Breast cancer is the most frequently diagnosed cancer in women (1). The treatment usually includes surgery, radiation therapy, oral or intravenous anticancer drugs, hormone therapy and targeted biological antibodies (2). In Clinical, it is usually classified into four subtypes: Luminal A, Luminal B, HER-2 overexpression, and triple negative breast cancer based on the expression of ER, PR, and HER-2 (3). Due to high heterogeneity of breast cancer, to identify other biomarkers may benefit the diagnosis and therapeutics.

In humans, there are two identified C5a receptors, C5aR1, known as CD88 likewise, and C5aR2, known as GPR77 or C5L2 likewise. Although the C5a–C5aR1 interaction is well-recognized as having proinflammatory and disease-inducing responses, the role of C5aR2 remains hotly debated (4, 5). Since C5AR2 was originally reported in 2000 (6), the last two decades have seen a quantity of studies reported that C5AR2 is accumulating attention for its unique role in dampening C5a signaling, modulating C5aR1 activity, and more recently, interplaying with other pattern recognition receptors and intracellular inflammasomes (7, 8). Reduced inflammatory cell infiltration was caused by deficiency of C5AR2, suggesting that C5AR2 had a critical effect on optimal C5a-mediated cell infiltration (9). The role of C5AR2 in properly controlling C5a is considerable; otherwise, excessive or unresolved C5a production can aggravate a plethora of acute and chronic diseases, such as ischemia-reperfusion injury, rheumatic arthritis, sepsis atherosclerosis, and cancer, even COVID-19 (10–13).

Research of C5AR2 in cancer is limited and controversial. It was reported a strong association of C5AR2 with chemoresistance and poor prognosis across diverse cohorts of patients with lung and breast cancer, together with IL-10 (14). On the contrary, in a melanoma bearing murine model, C5AR2 has a limited yet favorable effect in restricting tumor growth (15). In another AOM/DSS-induced CRC tumorigenesis, C5AR2 deficiency increased tumor progression, indicating that C5AR2 has an anti-inflammatory effect (16). However, the function and mechanism of C5AR2 independent of complement system in breast cancer remains unknown.

In this study, a comprehensive analysis was utilized to elucidate expression, prognosis, immune infiltration as well as correlation with signaling pathways of C5AR2. Immunohistochemistry of clinical samples and cell lines experiments were also conducted and the results were consistent with bioinformatics analysis. This present study may provide novel insights to show the potential of C5AR2 in breast cancer therapy.

## Material and Methods

### Data Processing and Analysis of C5AR2 Expression

The data of differential expression levels of C5AR2 between cancerous tissues and matched noncancerous tissue was from The Cancer Genome Atlas (TCGA, https://portal.gdc.cancer.gov/) and Genotype-Tissue Expression (GTEx, http://gtexportal.org) projects. The expression levels of C5AR2 in 31 normal tissues and 27 tumor tissues were evaluated, and the expression levels between cancerous samples and matched noncancerous ones were compared. Expression data were transformed by Log2and t-tests of two groups were performed for these types of tumor; P<0.05 was identified as a statistically significant difference between cancerous and noncancerous tissues.

### Survival Analysis

Univariate survival analysis was performed to illustrate the associations between C5AR2 expression and disease-free survival (DSS) in pan-cancer. Using a bipartite method, the expression levels of C5AR2 were distributed into two groups. The Kaplan-Meier plotter database (17) was utilized as well as the PrognoScan database (18). HR, 95% CI, and log-rank *P* values were calculated then displayed.

### Analysis of Immune Infiltration

The Tumor IMmune Estimation Resource (TIMER2.0, http://timer.cistrome.org/) is an integrated database designed to systematically analyze immune infiltrations and gene correlations across different tumor types (19). It provides the purity-adjusted spearman’s rho in diverse tumors, characterizing immune infiltrates’ abundances from the gene expression profiles estimated by CIBERSORT, QUANTISEQ, XCELL, and several other immune deconvolution multiple methods. The infiltrating levels of immune cells were compared between high and low C5AR2 expression cohorts in breast cancer.

### Correlation Analysis

To assess the correlations between C5AR2 and tumor mutational burden (TMB) as well as microsatellite instability (MSI), we conducted Spearman’s rank correlation coefficient, and the immune scores and gene correlation of each tumor sample were separately counted as well. Once P<0.05 and R>0.20, correlations were regarded as significantly positive.

### Enrichment Analysis

Kyoto Encyclopedia of Genes and Genomes (KEGG) is a powerful resource for understanding functions and utilities from molecular-level information. The molecular signatures database (MsigDB) was also demonstrated here for GSEA analysis, using the Hallmark gene set to illustrate specific biological states or processes (20). Once |NES|>1, *P*<0.05, FDR<0.25, pathways were regarded with significant enrichment. Meanwhile, GO analysis and GSVA analysis were conducted in breast cancer.

### Immunohistochemistry

Clinical samples of breast cancer and normal tissues were incubated with rabbit antibody against C5AR2 at 1:100 dilution at 4°C overnight. Then the sections were incubated with HRP-conjugated goat anti-rabbit IgG H&L at 1:400 dilution at room temperature for 1 hour. We used the GTVision III immunohistochemical detection kit to detect immunoreactivity. All fields were observed under the Olympus BX53 microscope. The difference was measured by the intensity and quantity.

### Cell Lines and Culture

MDA-MB-231, T47D, MCF7 cell lines were purchased from ATCC, then cultured in accordance with the manufacturer’s instructions. Cell transfection was conducted by lentiviral vector and screened by puromycin.

### RNA Extraction and qRT-PCR

NucleoZol reagent was used to isolate RNA from cells, and 2×SYBR PreMix EX TaqTM II was used to conduct qRT-PCR in accordance with the manufacturer’s instructions. The primer sequences were listed (5’-3’): C5AR2 forward - CTGCTGACCATGTATGCCAG, reverse- CGCTGAACCGTAGACCACC. β-actin forward- ACCGAGCGCGGCTACAG, reverse- CTTAATGTCACGCACGATTTCC. Results were calculated based on the 2–△△CT method.

### CCK8 Assay

Cells were plated in 96‐well plates, and during the following seven days, cell proliferation was measured daily by Cell Counting Kit-8 reagent in accordance with the manufacturer’s instructions. Using a microplate reader, the absorbance was measured at the indicated time at 450 nm.

### Transwell Assay

Fifty thousand breast cancer cells were seeded in the transwell, using the serum-free medium, and in the bottom 24-well plate, the medium with 10% fetal bovine serum was added. For invasion assay, diluted matrigel was pre-prepared. After incubation for 24 hours, cells on the upper membrane of the transwell were wiped off. Cells on the lower membrane of the transwell were fixed, stained, and then imaged and counted.

### Western Blot

Protein samples were quantified firstly and separated by page electrophoresis, then transferred to the special PVDF membranes. After membranes were blocked with 5% milk, then incubated with antibody. Signals were finally detected by chemiluminescence kit and imaged.

### Statistical Analysis

The Student’s t-test (two-tailed) was conducted in contrast between two groups. Spearman’s rank correlation test was used to obtain the P values and partial correlation values. Results with P<0.05 were considered as statistically significant, and significance is shown as *P<0.05, **P<0.01, ***P<0.001, and ****P<0.0001.

## Results

### Differential Expression of C5AR2 Between Samples of Tumor and Normal Tissues

**Figure 1:**
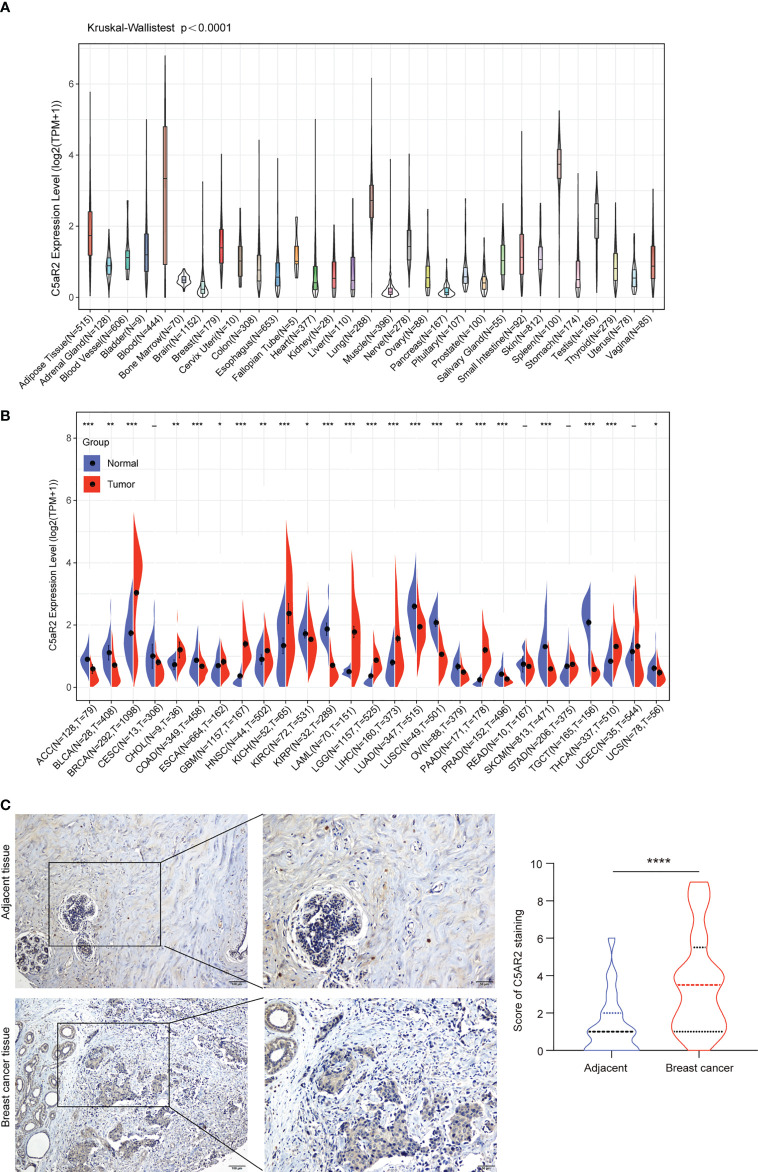
The expression levels of C5AR2 between cancerous and normal tissue samples. **(A)** C5AR2 expression in normal tissues. **(B)** The contrasts of C5AR2 expression between cancerous and noncancerous tissues from TCGA and GTEx data. **(C)** The representative images of C5AR2 staining in breast cancer and adjacent tissues. The protein expression levels of C5AR2 were detected by IHC staining in 45 paired breast cancer and adjacent tissues. Scale bar: 100 µm (left panel) or 50 µm (right panel). The quantitative results were shown in the right. *P < 0.05, **P < 0.01, ***P < 0.001, and ****P < 0.0001.

Physiologic C5AR2 expression was first evaluated across 31 normal tissues from the GTEx database (**Figure 1A**). It was in blood and spleen tissues that C5AR2 expression levels were the highest. However, they were quite lower in most other normal tissues. To figure out correlations of C5AR2 expression with cancer, we then evaluated and compared C5AR2 expression levels between different cancers and matched noncancerous samples. Results from the TCGA and GTEx database indicated that C5AR2 mRNA expression levels were dramatically elevated in BRCA, CHOL, ESCA, GBM, HNSC, KICH, LAML, LGG, LIHC, PAAD, and THCA tissues, while lower in ACC, BLCA, COAD, KIRC, KIRP, LUAD, LUSC, OV PRAD, SKCM, TGCT and UCS tissues confronted with that in normal ones (**Figure 1B**). Further, the protein expression levels were detected by immunohistochemical (IHC) staining in 45 paired breast cancer tissues. The results also showed higher C5AR2 expression level in the breast cancer tissues than the paired adjacent tissues (**Figure 1C**).

Notably, C5AR2 expression levels were diverse in subtypes of breast cancer, and it was much higher in Lumina A and Lumina B (ER-positive) than in HER2 and Basal (ER-negative) in the TIMER2.0 database (**Figure S1**). In addition, the comparisons of C5AR2 mRNA expression between the paired cancerous and noncancerous samples from the TCGA database were exhibited in **Figure S2**. C5AR2 mRNA expression levels were elevated in BRCA, HNSC, LIHC, PCPG, STAD, and THCA. By contrast, C5AR2 mRNA expression levels were declined in BLCA, KIRC, KIRP, LUSC, and THYM. In conclusion, C5AR2 was highly expressed in BRCA, CHOL, ESCA, GBM, HNSC, KICH, LAML, LGG, LIHC, PAAD, PCPG, STAD, and THCA, indicating C5AR2 as a potential tumor target.

### Prognostic Value of C5AR2 in Breast Cancer

**Figure 2:**
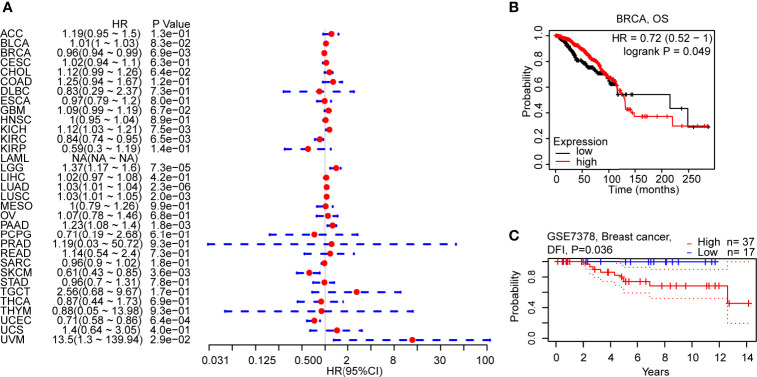
Correlations of C5AR2 expression with patient prognosis. **(A)** Forest plot of correlations between C5aR2 expression level and DSS across diverse tumors in TCGA database. **(B)** Survival curve of OS in BRCA in Kaplan-Meier plotter database. **(C)** Survival curve in the cohorts of GSE7378 in PrognoScan database.

To figure out how C5AR2 expression correlates with patient prognosis, survival analysis for diverse cancer types from the TCGA database was respectively conducted. Cox proportional hazards model analysis suggested the significant associations between C5AR2 expression and disease-specific survival (DSS) in breast cancer and several other cancer types (**Figure 2A**). Kaplan-Meier survival curves from the Kaplan-Meier plotter database showed that C5AR2 expression levels also had significant associations with overall survival (OS) in several cancer types (**Figure S3**), including BRCA (**Figure 2B**). Considering that C5AR2 expression levels were much higher in Lumina A and Lumina B (ER-positive) than in HER2 and Basal (ER-negative), the PrognoScan database was used as well. The cohort GSE7378 included 100% ER-positive breast cancer samples suggested that elevated C5AR2 expression levels were significantly correlated with poorer DFI in ER-positive breast cancer (**Figure 2C**). In a word, high C5AR2 expression was associated with a poorer prognosis in BRCA, especially the ER-positive breast cancer, in which C5AR2 expression levels were more elevated than in normal tissues, indicating C5AR2 as an oncogene.

### Correlations Between C5AR2 Expression and Immune Infiltration, TMB, and MSI

DNA mismatch repair deficiency (MMRd) frequently leads to microsatellite instability-high (MSI-H), then results in the aggravation of tumor mutation burden (TMB). These hypermutation elements contribute to tumorigenesis and are considered as independent predictors of immune checkpoint blockade effectiveness (21, 22). Via Spearman’s rank correlation coefficient, associations of TMB and MSI with C5AR2 expression were separately analyzed in pan-cancer. The result revealed negative associations in BRCA and GBM (**Figure S4A**). C5aR2 expression levels were negatively related to MSI in BRCA and STAD (**Figure S4B**).

**Figure 3:**
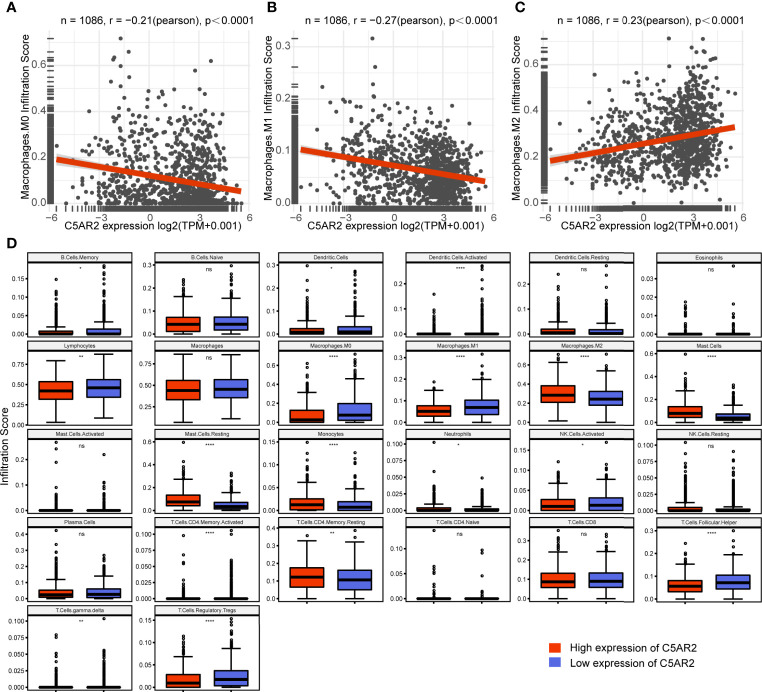
Relationships of C5AR2 expression to immune infiltration in breast cancer. **(A–C)** Relationships of C5AR2 expression to the infiltration scores of macrophages. **(D)** The infiltration levels of 26 tumor-infiltrating immune cells compared in the high and low C5AR2 expression groups in breast cancer. NS, not significant, *P < 0.05, **P < 0.01, and ****P < 0.0001.

To explore how C5AR2 expression influences immune infiltration, we used the TIMER2.0 database to exhibit the heatmap of correlations of C5AR2 expression levels with various immune infiltrates, including macrophages, monocytes, neutrophils, dendritic cells (DCs), and regulatory T cells (Tregs) in pan-cancer (**Figure S5**). Notably, in breast cancer, C5AR2 expression levels were positively related to immune infiltration of M2 macrophages while negatively related to M0 and M1 macrophages (**Figures 3A–C**). Moreover, the infiltration scores of diverse immune cells in breast cancer patients were evaluated. The infiltration scores of M0 and M1 macrophages were lower, while that of M2 macrophages were elevated in the group of high C5AR2 expression than those in the group of low C5AR2 expression (**Figure 3D**). The results indicated that C5AR2 actively participated in immune infiltration, especially the polarization of macrophages.

### Enrichment Analysis

**Figure 4:**
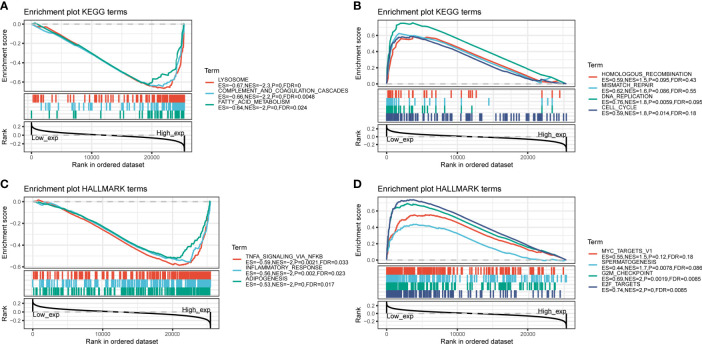
GSEA analysis of C5AR2 related to signaling pathways and biological states or processes in KEGG and hallmark datasets. **(A)** The top three rankings of signaling pathways in the KEGG dataset by samples of high C5AR2 expression. **(B)** The top three rankings of signaling pathways in the KEGG dataset with the low C5AR2 expression. **(C)** The top three rankings of signaling pathways in the hallmark dataset by the high C5AR2 expression sample. **(D)** The top three rankings of signaling pathways in the hallmark dataset with low C5AR2.

To investigate how C5AR2 expression impact the fate of tumors, GSEA analysis was conducted, dividing the pan-cancer samples into high expression group and low one based on the C5AR2 expression levels, and in separately high and low expression groups, then analyzing the enrichment of signaling pathways or biological states or processes in both KEGG and hallmark datasets. Ranked by NES score, the top fifteen most abundant signaling pathways or biological processes have been listed and demonstrated in **Tables S1** and **S2**, and the top three were shown in **Figure 4**. The results indicated that C5AR2 positively regulates apoptosis, lysosome, peroxisome, fatty acid metabolism, glycosaminoglycan degradation, and other biological processes of KEGG signaling pathways. In hallmark signaling pathways, TNFα signaling *via* NFκB, IL6 JAK STAT3 signaling, IL2 STAT5 signaling, inflammatory response, KRAS signaling up, reactive oxygen species pathway, p53 pathway, and apoptosis were considered as the most enriched. Taken together, C5AR2 widely participated in regulating tumor immunity and metabolic signaling pathways.

**Figure 5:**
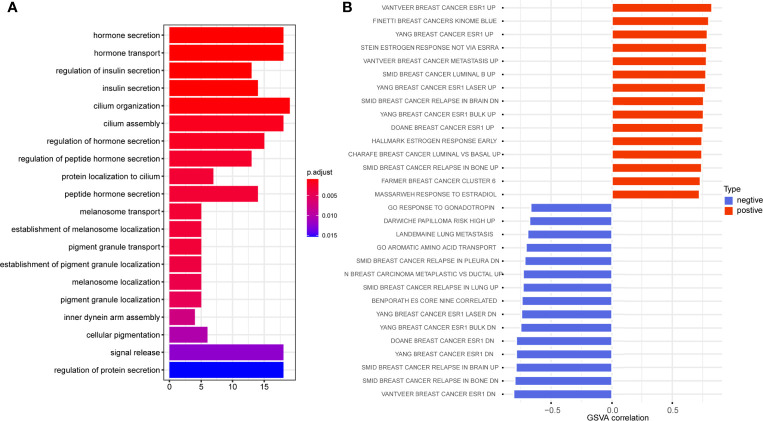
GO enrichment analysis **(A)** and GSVA analysis **(B)** of C5AR2 related to biological processes in breast cancer.

To further figure out the biological significance of C5AR2 in breast cancer, GO enrichment analysis of the biological process was conducted, and it suggested that C5AR2 was associated mainly with hormone secretion and transport (**Figure 5A**). We also performed GSVA analysis, and the results revealed that C5AR2 was notably associated with metastasis as well as relapse of breast cancer and the upregulation of ESR1, a proven oncogene in breast cancer (**Figures 5B**).

### C5AR2 Overexpression Facilitated the Malignant Behaviors and Oncogenic Signaling in Breast Cancer Cells

**Figure 6:**
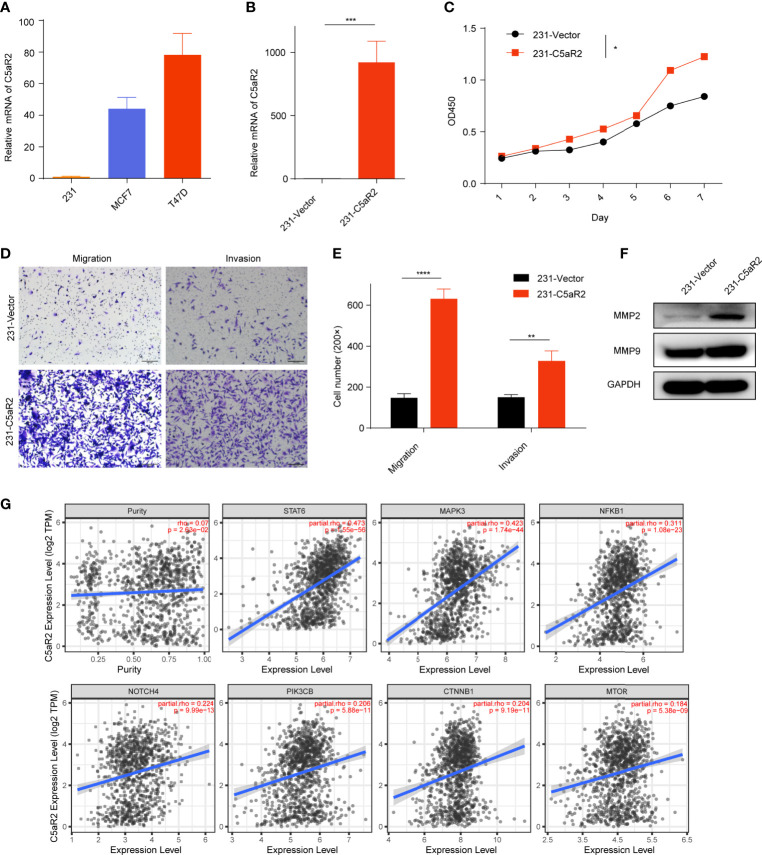
Overexpression of C5AR2 facilitated the migration, invasion, and proliferation in breast cancer cells. **(A)** C5AR2 expression levels were measured in various breast cancer cell lines through qRT-PCR. **(B)** MDA-MB-231 cells were transfected with C5AR2 overexpressed plasmid, and the level of C5AR2 was measured through qRT-PCR. **(C)** The proliferation of MDA-MB-231 cells was measured through CCK8 assay. **(D, E)** The migration and invasion of MDA-MB-231 cells were measured through transwell assay. **(F)** MMP2 and MMP9 expression levels were measured through Western Blot in control and C5AR2 overexpressed MDA-MB-231 cells. **(G)** Correlation between C5AR2 and certain classic genes in key signaling pathways in breast cancer through TIMER database. *P < 0.05, **P < 0.01, ***P < 0.001, and ****P < 0.0001.

C5AR2 expression was most enriched in breast cancer tissues (**Figure 1B**), which was consistent with the results showed in the CCLE database (https://portals.broadinstitute.org/ccle/page?gene=C5AR2), C5AR2 expression levels were highest in a few breast cancer cell lines among solid tumor ones. Then C5AR2 expression levels in multiple breast cancer cell lines were evaluated, and compared with that in T47D and MCF7cells (ER-positive), C5AR2 expression was relatively lower in MDA-MB-231 cells (ER-negative) (**Figure 6A**). To explore how C5AR2 affects the proliferation of breast cancer cells, we overexpressed C5AR2 in MDA-MB-231 cells and validated the success of C5AR2 overexpression in this cell line (**Figure 6B**). The proliferation rates of MDA-MB-231 cells were significantly promoted following C5AR2 overexpression as evidenced by the CCK8 assay (**Figure 6C**). Besides, transwell assay was performed, and the results revealed that C5AR2 overexpression also significantly enhanced the migratory and invasive capacity of breast cancer cells (**Figure 6D**). Finally, we conducted Western Blot, and the results suggested that in MDA-MB-231 cells, C5AR2 overexpression led to the obviously upregulated levels of MMP2 and MMP9 (**Figure 6E**), indicating that C5AR2 was related to EMT in breast cancer. In addition, the relationships of C5AR2 to other classic genes in key signaling pathways in breast cancer were analyzed through the TIMER database (**Figure 6F**). As shown in **Figure 6G**, C5AR2 expression levels were significantly, strongly, and positively related to MAPK3, STAT3, and NFKB1 and moderately positively related to the three other genes (PIK3CB, CTNNB1, MTOR). Overall, C5AR2 promotes the proliferation, migration, invasion, and activation of oncogenic pathways in breast cancer cells.

## Discussion

As a target for therapeutic intervention, the complement cascade is becoming increasingly attractive to numerous academic and pharmacy corporations that have focused on projects that exploit this system to discover new drugs in inflammatory disorders (23). Although C5aR1 is highly regarded as pro-inflammatory and pathogenic in a multitude of inflammatory diseases and C5AR2 is also involved in those inflammatory diseases, including gout, sepsis, hidradenitis suppurativa, and type 2 diabetes, the nature of C5AR2 appears to be much more nuanced and multifaceted (24–27). More and more discoveries have made it clear that complement proteins exist in the tumor environment and impact tumor progress (28). Analyze of C5AR2 in cancer is scarce and mostly focused on knockout mice models. Here we first demonstrated its profiles of expression, prognosis, immune infiltration, malignant properties, and functional signaling in breast cancer.

In this present study, expression levels of C5AR2 in different cancer types and normal samples were evaluated using the TCGA and GTEx databases, indicating that in pan-cancer, there were distinct differences of C5AR2 expression across cancerous and normal tissues. C5AR2 expression was increased in BRCA, CHOL, ESCA, GBM, HNSC, KICH, LAML, LGG, LIHC, PAAD, PCPG, STAD, and THCA, while decreased in ACC, BLCA, COAD, KIRC, KIRP, LUAD, LUSC, OV PRAD, SKCM, TGCT, THYM, and UCS confronted with adjacent normal controls. Notably, C5AR2 expression levels were much higher in Lumina A and Lumina B (ER-positive) than in HER2 and Basal (ER-negative). Strong correlations were shown between elevated C5AR2 expression and poorer prognosis in BRCA (ER-positive), indicating that C5AR2 has a malignant biological character as well as specific prognostic value, and it may be an oncogene in breast cancer.

One more important discovery is the relationships of C5AR2 expression to immune infiltration. C5AR2 was mainly expressed on macrophages, e.g. Kuffer cells, and mesenchymal cells, e.g. Ito cells in the HPA database (https://www.proteinatlas.org/ENSG00000134830-C5AR2/celltype), then the abundance of these two immune cells infiltrating in the tumor microenvironment may be indirectly reflected by C5AR2 expression levels. In this study, C5AR2 expression showed remarkable relationships to immune infiltrating levels of multiple immune cells, especially the CAFs and macrophages. A previous study revealed that in primary human macrophages, C5AR2 possessed pleiotropic functions (29). In this study, we noticed that C5AR2 was involved in the polarization of macrophages, and C5AR2 expression was positively associated with M2 macrophages and negatively with M1 macrophages in breast cancer. We analyzed the relationships of C5AR2 expression levels to TMB and MSI as well, and the results suggested that C5AR2 might have a synergy effect with known immune checkpoints. Nevertheless, according to TISIDB (30), several published reports were summarized (http://cis.hku.hk/TISIDB/browse.php?gene=C5AR2), and no significant difference in C5AR2 expression levels was found between responders and non-responders to immunotherapy. Taken together, we supposed that the promotion of the polarization of M2 macrophages by C5AR2 leads to an accelerative effect in tumor initiation or development in breast cancer.

Meanwhile, GSEA analysis was performed, revealing that C5AR2 was widely involved in metabolic pathways and biosynthesis in pan-cancer, including TNFα signaling *via* NFκB, IL6 JAK STAT3 signaling, IL2 STAT5 signaling, inflammatory response, KRAS signaling up, reactive oxygen species pathway, p53 pathway, and apoptosis. In this present report, the gene co-expression analysis revealed the relationship of C5AR2 expression to the activation of oncogenic signaling such as NF-kB. And a previous study revealed that persistent NF-kB activation was maintained by complement signaling *via* C5AR2 (14). GO analysis in breast cancer was conducted, and the results revealed the association between C5AR2 and hormone secretion and transport. Since endocrine therapy is one of the basic methods for treating hormone receptor-positive breast cancer, C5AR2 may play a role in curative effect (1). We used GSVA analysis as well and noticed that C5AR2 was significantly associated with metastasis as well as relapse of breast cancer and the upregulation of ESR1, a proven oncogene in breast cancer involved in endocrine resistance (31).

Then we demonstrated cell experiments to confirm C5AR2 facilitates the migration, invasion, and proliferation in breast cancer cells. C5AR2 expression levels were higher in T47D and MCF7cells (ER-positive) than in MDA-MB-231 cells (ER-negative). Here, C5AR2 overexpression in MDA-MB-231 cells was performed, which promoted migration, invasion, and proliferation. C5AR2 overexpression also upregulated the expression levels of MMP2 and MMP9, which were reported as oncogenes correlated with metastasis and invasion in various cancers (32). MMP2 also activates TGF-β to promote epithelial-mesenchymal transformation (EMT), while by releasing vascular endothelial growth factor (VEGF), MMP9 promotes tumor angiogenesis (33, 34). The results indicated the role of C5AR2 in the metastasis and invasion of breast cancer.

There are some shortcomings and inadequacies in the present study. Firstly, this complex analysis has been done for the first time and no good comparable data is available. Moreover, if experimental validation of C5AR2 knockdown in T47D or MCF7 cells is performed as well, the hypothesis will be more convincing, together with the experiments of C5AR2 overexpression in MDA-MB-231 cells.

In summary, our present study provides insights into the malignant properties of C5AR2 and its potential role in tumor immunology, suggesting that C5AR2 can stand as a prospective biomarker in breast cancer.

## Data Availability Statement

The datasets presented in this study can be found in online repositories. The names of the repository/repositories and accession number(s) can be found in the article/**Supplementary Material**.

## Author Contributions

YZ, XW, and YX conceived of the presented idea and carried out the experiments. YZ and LC performed the analytic calculations. YZ wrote the initial manuscript. PD and JC reviewed the manuscript. WH supervised all the work. All authors contributed to the article and approved the submitted version.

## Funding

This work was supported by grants to W.H. from the National Natural Science Foundation of China (82121004, 81790254, 91629301 and 81872354) and the Major State Basic Research Development Program of China (2013CB910802).

## Conflict of Interest

The authors declare that the research was conducted in the absence of any commercial or financial relationships that could be construed as a potential conflict of interest.

## Publisher’s Note

All claims expressed in this article are solely those of the authors and do not necessarily represent those of their affiliated organizations, or those of the publisher, the editors and the reviewers. Any product that may be evaluated in this article, or claim that may be made by its manufacturer, is not guaranteed or endorsed by the publisher.
