# Supplementary figures and images for: An Integrated Analysis of C5AR2 Related to Malignant Properties and Immune Infiltration of Breast Cancer
Source: Front Oncol. 2021 Sep 14;11:736725. doi: 10.3389/fonc.2021.736725 (PMC8476960; doi:10.3389/fonc.2021.736725)

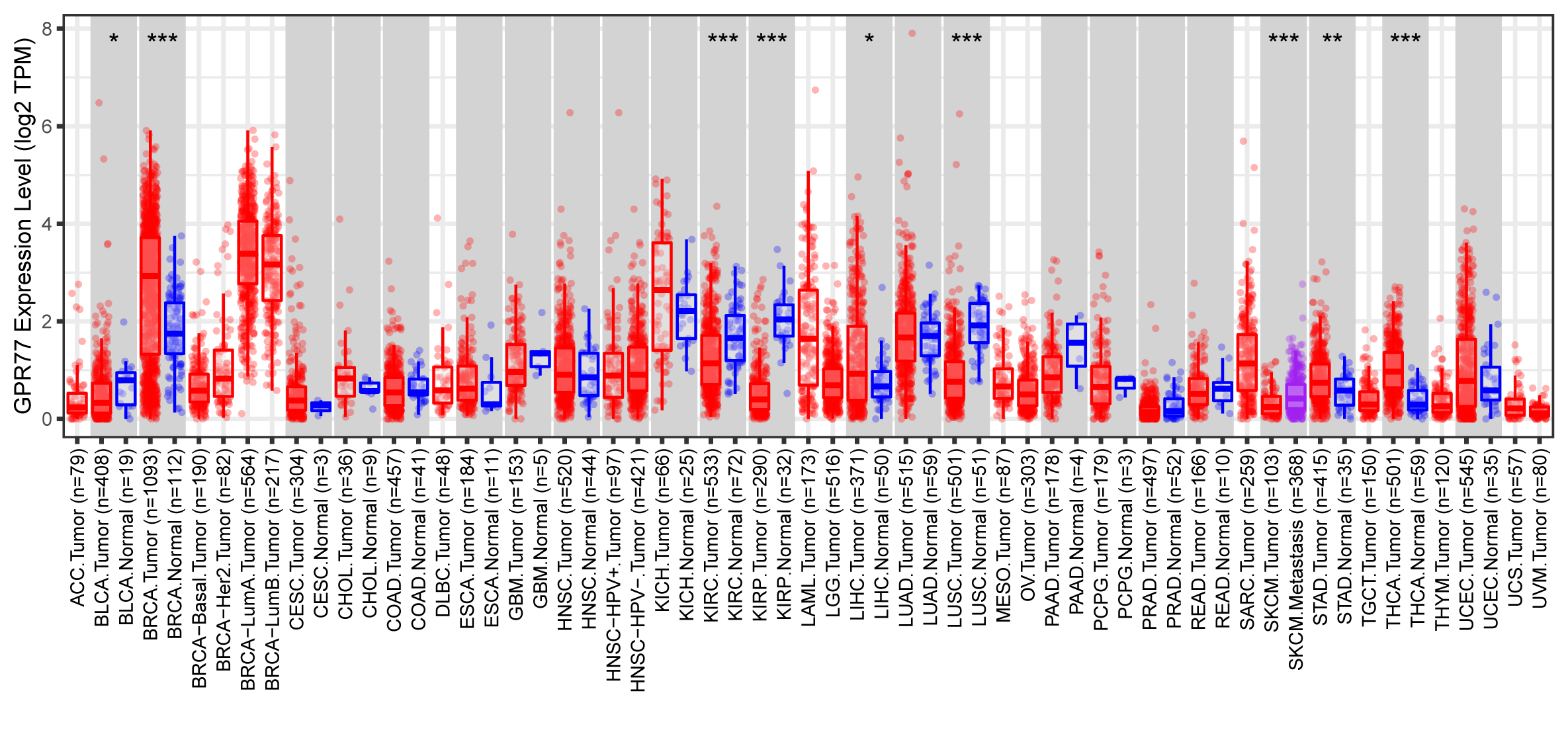

Supplement: Supplementary file 1 [file Image_1.tif]

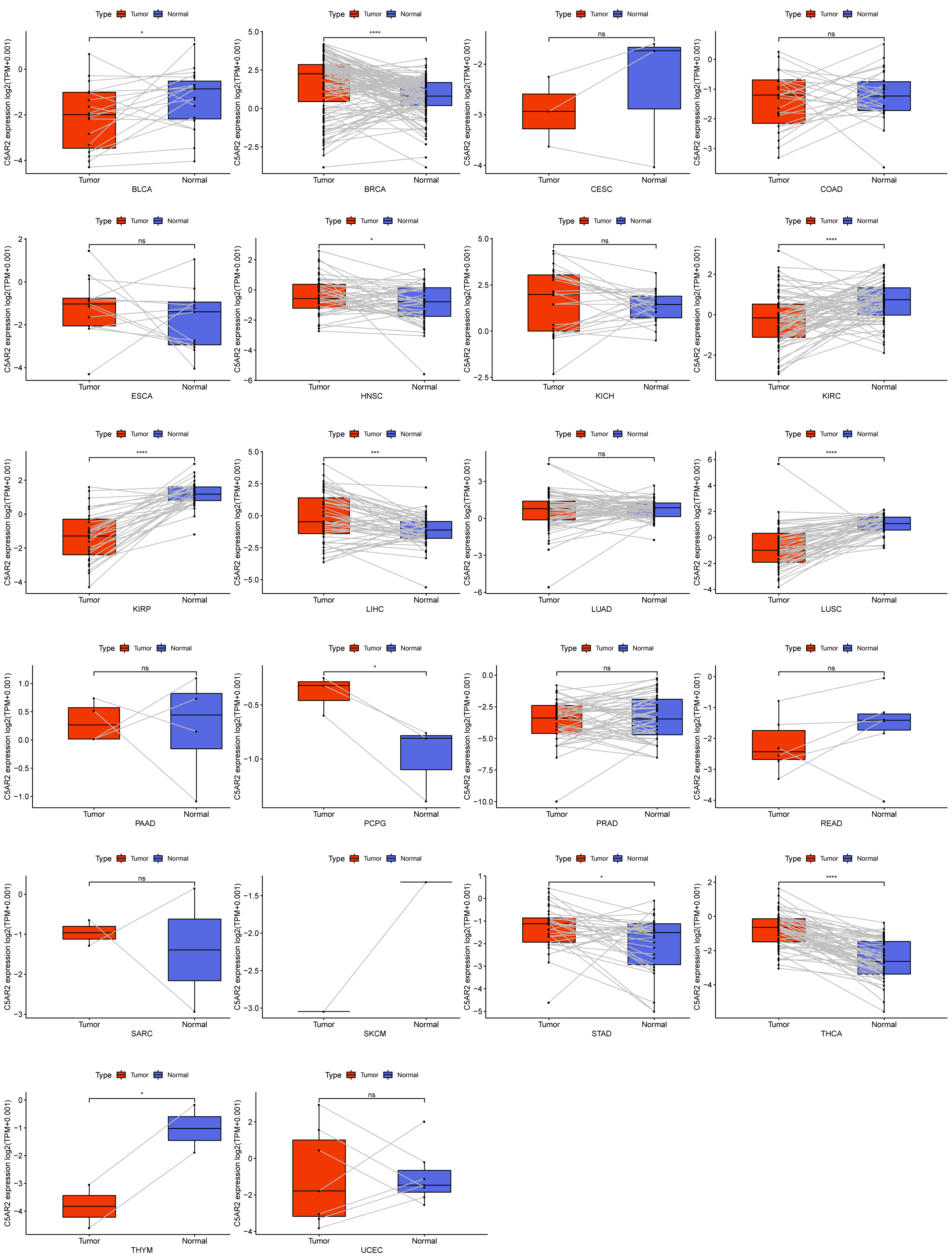

Supplement: Supplementary file 2 [file Image_2.tif]

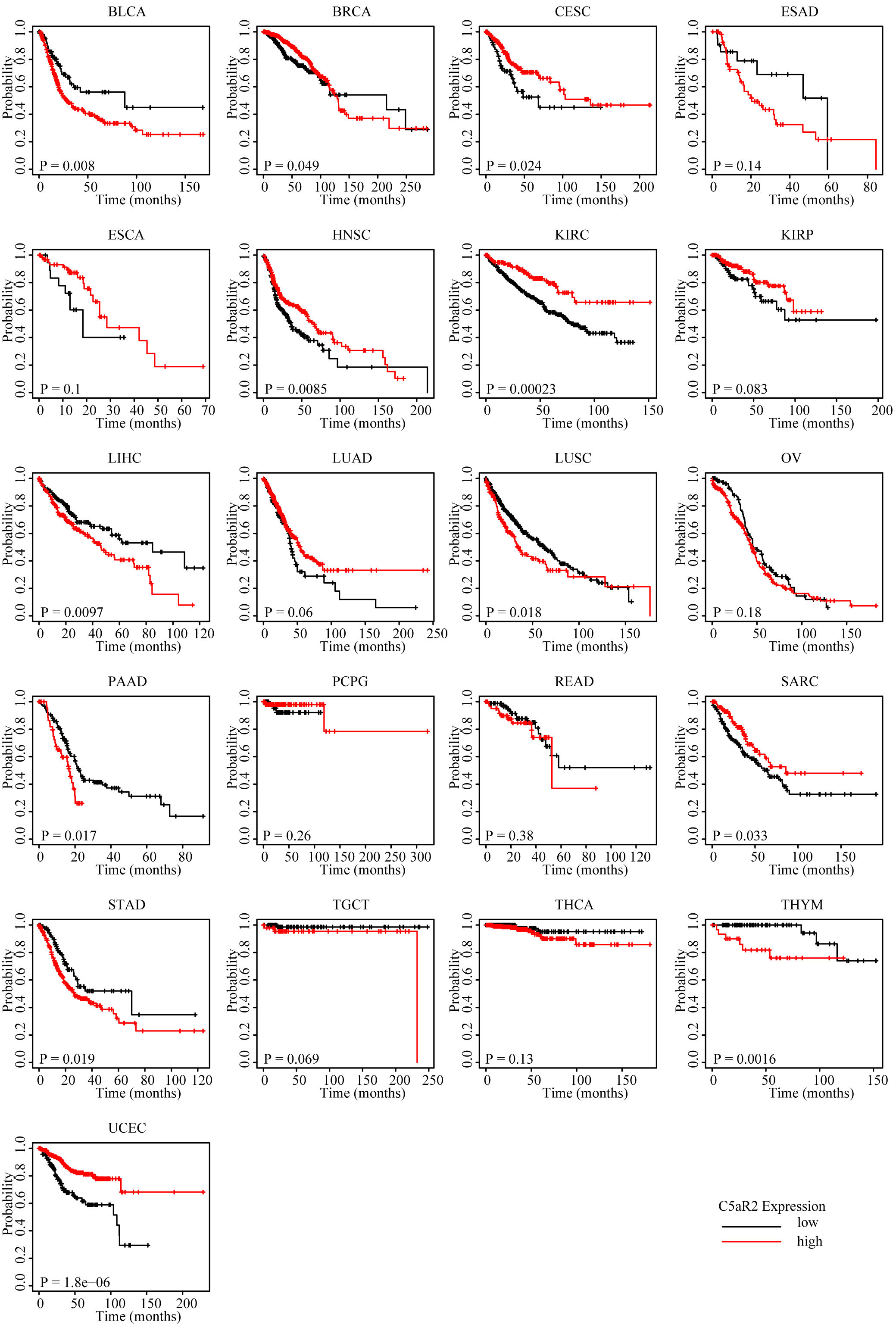

Supplement: Supplementary file 3 [file Image_3.tif]

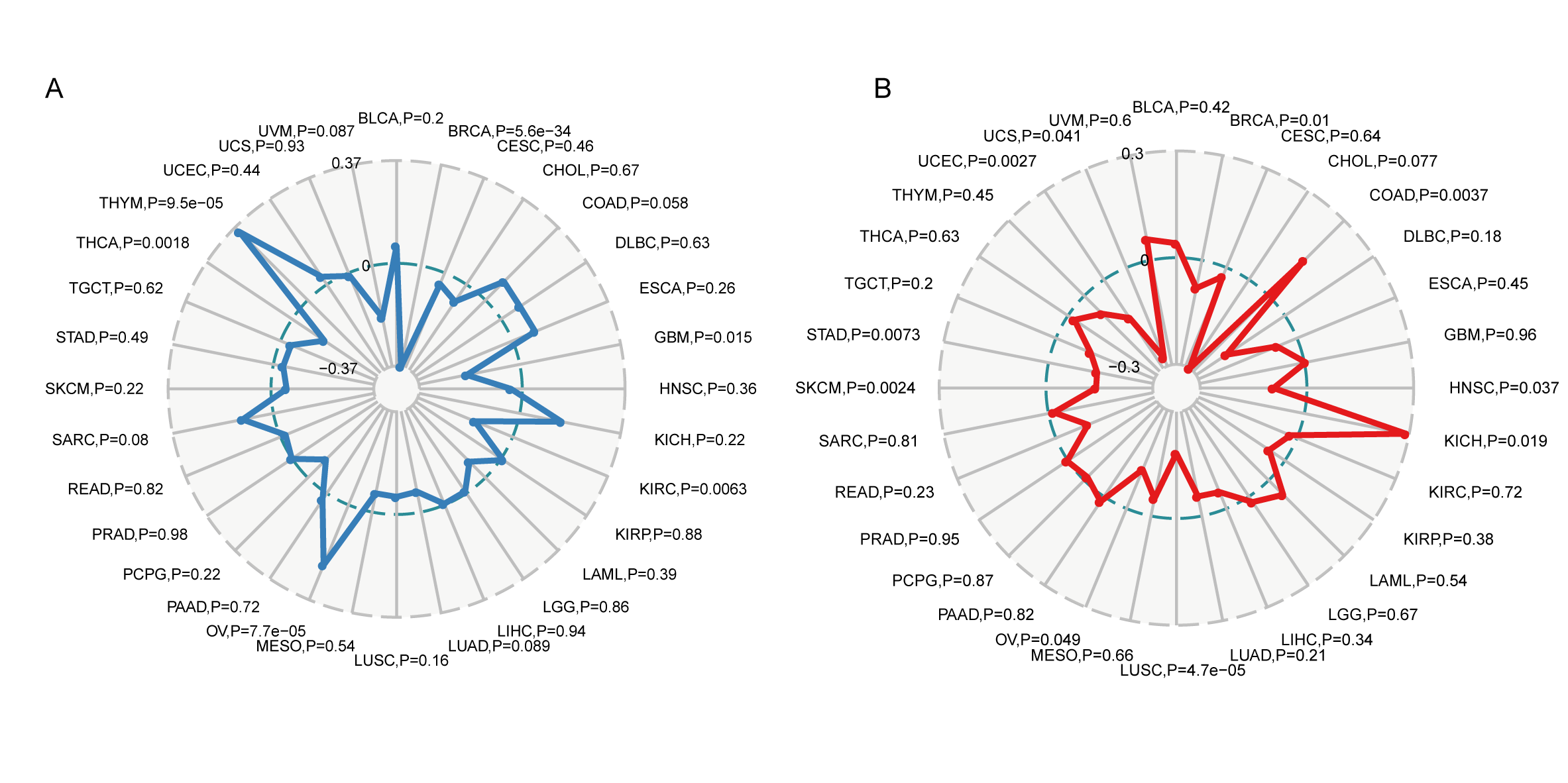

Supplement: Supplementary file 4 [file Image_4.tif]

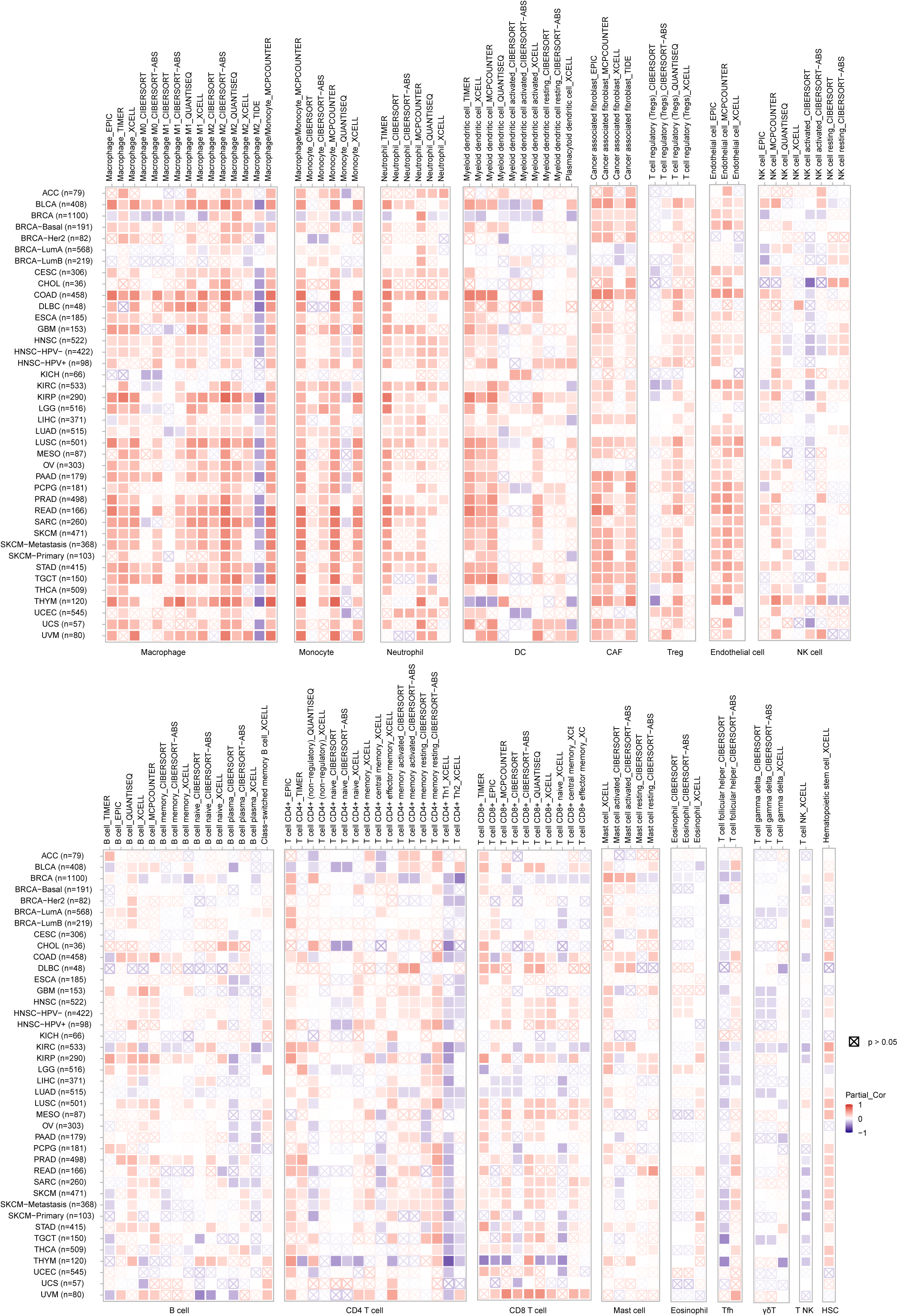

Supplement: Supplementary file 5 [file Image_5.tif]
