# Supplementary material for: An Integrated Analysis of C5AR2 Related to Malignant Properties and Immune Infiltration of Breast Cancer
Source: Front Oncol. 2021 Sep 14;11:736725. doi: 10.3389/fonc.2021.736725 (PMC8476960; doi:10.3389/fonc.2021.736725)
Supplement: Supplementary file 6 [file Table_1.docx]

Supplementary Material

**Supplementary Table 1.** KEGG terms of top 15 GSEA analysis.

| Term | ES | NES | NP | FDR | FWER |
| --- | --- | --- | --- | --- | --- |
| KEGG_LYSOSOME | -0.6723 | -2.3072 | 0 | 0 | 0 |
| KEGG_COMPLEMENT_AND_COAGULATION_CASCADES | -0.6568 | -2.2007 | 0 | 0.0048 | 0.005 |
| KEGG_FATTY_ACID_METABOLISM | -0.644 | -2.047 | 0 | 0.0239 | 0.038 |
| KEGG_ADIPOCYTOKINE_SIGNALING_PATHWAY | -0.5455 | -2.0344 | 0 | 0.0227 | 0.043 |
| KEGG_LEISHMANIA_INFECTION | -0.672 | -2.0285 | 0.0058 | 0.0198 | 0.046 |
| KEGG_ARACHIDONIC_ACID_METABOLISM | -0.5525 | -2.0281 | 0 | 0.0172 | 0.048 |
| KEGG_GLYCOSAMINOGLYCAN_DEGRADATION | -0.7005 | -2.0247 | 0 | 0.0152 | 0.049 |
| KEGG_GLYCOSPHINGOLIPID_BIOSYNTHESIS_GANGLIO_SERIES | -0.7227 | -2.0059 | 0 | 0.0167 | 0.06 |
| KEGG_AMINO_SUGAR_AND_NUCLEOTIDE_SUGAR_METABOLISM | -0.5887 | -1.9707 | 0 | 0.0214 | 0.081 |
| KEGG_OTHER_GLYCAN_DEGRADATION | -0.7489 | -1.9415 | 0.0061 | 0.0268 | 0.105 |
| KEGG_PPAR_SIGNALING_PATHWAY | -0.5229 | -1.9326 | 0 | 0.0283 | 0.116 |
| KEGG_NOD_LIKE_RECEPTOR_SIGNALING_PATHWAY | -0.5654 | -1.8785 | 0.002 | 0.0469 | 0.187 |
| KEGG_PEROXISOME | -0.5236 | -1.8686 | 0.006 | 0.0471 | 0.2 |
| KEGG_APOPTOSIS | -0.4992 | -1.8607 | 0.0079 | 0.0476 | 0.213 |
| KEGG_NICOTINATE_AND_NICOTINAMIDE_METABOLISM | -0.5662 | -1.8599 | 0 | 0.0446 | 0.213 |
