# Supplementary material for: An Integrated Analysis of C5AR2 Related to Malignant Properties and Immune Infiltration of Breast Cancer
Source: Front Oncol. 2021 Sep 14;11:736725. doi: 10.3389/fonc.2021.736725 (PMC8476960; doi:10.3389/fonc.2021.736725)
Supplement: Supplementary file 7 [file Table_2.docx]

Supplementary Material

**Supplementary Table 2.** HALLMARK terms of top 15 GSEA analysis.

| Term | ES | NES | NP | FDR | FWER |
| --- | --- | --- | --- | --- | --- |
| HALLMARK_TNFA_SIGNALING_VIA_NFKB | -0.5892 | -2.0233 | 0.0021 | 0.0328 | 0.02 |
| HALLMARK_INFLAMMATORY_RESPONSE | -0.5597 | -1.9972 | 0.002 | 0.0234 | 0.027 |
| HALLMARK_ADIPOGENESIS | -0.5292 | -1.991 | 0 | 0.0174 | 0.031 |
| HALLMARK_HEME_METABOLISM | -0.4635 | -1.9524 | 0 | 0.0222 | 0.051 |
| HALLMARK_IL6_JAK_STAT3_SIGNALING | -0.5799 | -1.9029 | 0.0119 | 0.0273 | 0.073 |
| HALLMARK_IL2_STAT5_SIGNALING | -0.4571 | -1.8835 | 0.0042 | 0.0269 | 0.084 |
| HALLMARK_XENOBIOTIC_METABOLISM | -0.4544 | -1.8806 | 0.0041 | 0.0235 | 0.084 |
| HALLMARK_COAGULATION | -0.5016 | -1.8308 | 0.0041 | 0.0324 | 0.118 |
| HALLMARK_COMPLEMENT | -0.4831 | -1.8237 | 0.0042 | 0.0298 | 0.122 |
| HALLMARK_BILE_ACID_METABOLISM | -0.4477 | -1.8075 | 0.0065 | 0.0317 | 0.146 |
| HALLMARK_FATTY_ACID_METABOLISM | -0.4698 | -1.7439 | 0.0236 | 0.0474 | 0.2 |
| HALLMARK_REACTIVE_OXYGEN_SPECIES_PATHWAY | -0.5551 | -1.7314 | 0.0129 | 0.0464 | 0.21 |
| HALLMARK_KRAS_SIGNALING_UP | -0.4274 | -1.7286 | 0.0168 | 0.0434 | 0.213 |
| HALLMARK_P53_PATHWAY | -0.4228 | -1.6852 | 0.0192 | 0.0601 | 0.272 |
| HALLMARK_APOPTOSIS | -0.4131 | -1.6243 | 0.0361 | 0.0813 | 0.343 |
